# Third-Generation Cephalosporin–Resistant *Vibrio cholerae*, India

**DOI:** 10.3201/eid1808.111686

**Published:** 2012-08

**Authors:** Jharna Mandal, Vilwanathan Sangeetha, Vithiya Ganesan, Mohamudha Parveen, Venkatesan Preethi, Belgode Narasimha Harish, Sampath Srinivasan, Subhash Chandra Parija

**Affiliations:** Jawaharlal Institute of Postgraduate Medical Education and Research, Puducherry, India

**Keywords:** Vibrio cholerae, ceftriaxone resistance, blaDHA-1, blaNDM-1, JIPMER, bacteria, India, antimicrobial rresistance, ESBL, extended-spectrum beta-lactamase

## Abstract

*Vibrio cholerae* resistance to third-generation cephalosporins is rarely reported. We detected a strain that was negative for extended-spectrum β-lactamase and positive for the AmpC disk test, modified Hodge test, and EDTA disk synergy test and harbored the *blaDHA-1* and *blaNDM-1* genes. The antimicrobial drug susceptibility profile of *V. cholerae* should be monitored.

*Vibrio cholerae* has developed enormous capabilities to combat antimicrobial drug effect. It possesses efflux pumps that act on multiple classes of antimicrobial drugs and elaborates enzymes that can hydrolyze complex antimicrobial drugs. It also can share antimicrobial resistance genes through integrons and conjugative plasmids that enable easy transfer of antimicrobial drug resistance genes and thus contribute to spread of antimicrobial resistance (*1*).

Even though fluid replacement plays a major role in treating cholera during outbreaks, antimicrobial drugs are crucial for controlling the disease and its spread. Antimicrobial therapy reduces shedding of the *Vibrio cholerae* bacillus in feces from >5 days to 1–2 days, thereby reducing the volume of diarrheic stool and the duration of illness, hastening recovery, and decreasing the chances of disease spread. In the absence of effective antimicrobial therapy, infected persons shed the bacillus for >5 days. Reported resistance to most commonly used antimicrobial drugs, i.e., tetracycline and ciprofloxacin (*1*), has limited options for therapy. Such drug-resistant *V. cholerae* strains threaten public health (*2*). Resistance to third-generation cephalosporins has rarely been reported (*1*). Our goal was to determine the mechanism(s) of resistance to third-generation cephalosporin by phenotypic and genotypic methods.

## The Study

A 2-year-old child was admitted to the department of pediatrics at the Jawaharlal Institute of Postgraduate Medical Education and Research (JIPMER), Puducherry, India, with a provisional diagnosis of cholera. Before the child received antimicrobial therapy, a fecal specimen was submitted to the Department of Microbiology at JIPMER. The child was treated successfully with intravenous fluid supplements and ciprofloxacin.

Meanwhile, a strain of *V. cholerae* O1 El Tor Ogawa was isolated from the fecal specimen. This strain was biochemically identified (*3*) and confirmed by agglutination with specific antiserum (BD Difco, Becton Dickinson, Sparks, MD, USA). Antimicrobial drug susceptibility testing was conducted by Kirby–Bauer method in accordance with the Clinical and Laboratory Standards Institute (*4*) against ampicillin (10 μg), ceftriaxone (30 μg), ciprofloxacin (5 μg), furoxone (300 μg), cotrimoxazole (25 μg), and tetracycline (30 μg). The MIC for ceftriaxone was determined by the agar dilution method and Etest for the El Tor Ogawa strain. For the agar dilution method, we doubled dilutions of ceftriaxone sodium (Himedia, Mumbai, India) from 0.5 μg/mL to 128 μg/mL, which included the recommended break points (*5*). American Type Culture Collection *Escherichia coli* 25922 was spread on each plate as growth control. The Etest was performed according to the manufacturers’ instructions (AB bioMérieux, Solna, Sweden). We performed the combination disk test using cefotaxime and ceftazidime, alone and in combination with clavulanic acid, to detect extended-spectrum β-lactamase (ESBL) in accordance with Clinical and Laboratory Standards Institute guidelines (*4*). The AmpC disk test was performed as described (*6*). The modified Hodge test and imipenem-EDTA disk synergy test for the phenotypic detection of carbapenemase were performed as described (*4**,**7*). We extracted DNA from the strain by using the boiling method. PCR was performed to detect ESBL genes, i.e., *blaCTX* (*8*), *blaSHV* (*8*), and *blaTEM* (*9*); a multiplex PCR was used to detect the AmpC group of genes, i.e., *blaMOX*, *blaCIT*, *blaDHA*, and *blaACC* (*10*). A multiplex PCR to detect carbapenemase genes, i.e., *blaKPC*, *blaIMP*, *blaVIM*, and *blaNDM*, was performed (*11*). Sequencing was performed to identify AmpC β-lactamase gene and the carbapenemase gene. Sequencing of these genes was conducted by Macrogen Inc. (Seoul, South Korea). We used the BLASTN program (www.ncbi.nlm.nih.gov/BLAST) for database searching.

The strain was resistant to ampicillin, ceftriaxone, cotrimoxazole, and furoxone and sensitive only to ciprofloxacin and tetracycline. The MIC for ceftriaxone was 16 μg/mL. The phenotypic tests for ESBL detection were negative. PCRs for detection of the ESBL genes were negative. Because the strain was negative for the phenotypic and the genotypic tests for ESBL production, in our attempt to determine the reason for the increased MIC, we further tested for the AmpC type of β-lactamase production. The strain, an AmpC β-lactamase producer, was positive for the *blaDHA* gene (405 bp) by multiplex PCR. On sequencing, the *blaDHA* gene detected had 99% identity with *Klebsiella pneumoniae* β-lactamase *blaDHA-1* gene (GenBank accession no. AY635140.1). Because carbapenems are considered the treatment of choice for AmpC-producing organisms, we tested the strain for the production of carbapenemase and found it to be a carbapenemase producer by a positive modified Hodge test and by the EDTA disk synergy test. The multiplex PCR for detection of carbapenemase genes yielded an amplicon of 660 bp, which was confirmed by sequencing to be an *blaNDM-1* gene and had 100% identity with *E. coli* strain HK-01 plasmid pNDM-HK (GenBank accession no. HQ451074.1).

To demonstrate the presence of a plasmid bearing the *blaNDM-1* gene, we isolated a plasmid using the alkaline lysis method (*12*) from the *V. cholerae* strain. This plasmid was subjected to the multiplex PCR to detect the carbapenemase genes mentioned earlier (*11*), which yielded an amplicon of 660 bp matching that of the *blaNDM-1* gene amplicon. We transferred this plasmid by a method described by Lee et al. (*13*) and used *E. coli* J53 as the recipient, which was isolated on meropenem (4 mg/L) containing Luria-Bertani agar and MacConkey agar, meropenem (4 mg/L), and sodium azide (100 mg/L) (*14*). We also subjected the *V. cholerae* plasmid to the multiplex PCR to detect the AmpC gene, as mentioned earlier, but none of the genes were amplified. We failed to transfer the AmpC gene (*blaDHA-1*) by using the plasmid transfer experiment. Such failures of transfer of *blaDHA-1* have been mentioned by Yan et al. (*15*).

Of the many AmpC genes known are 2 types of *blaDHA* genes: *blaDHA-1* and *blaDHA-2*; both are inducible plasmid-mediated genes. Because the strain reported here was already resistant to a third-generation cephalosporin, i.e., ceftriaxone, we did not perform the inducible AmpC test and proceeded to perform the AmpC disk test. Plasmid-mediated inducible β-lactamases are extremely rare and are most often constitutive (*15*). The AmpC disk test does not differentiate between chromosomal and plasmid-mediated AmpC β-lactamases. We do not have complete information about the chromosomal AmpC genes in *V. cholerae*.

Carbapenems are the treatment of choice for AmpC-producing strains (*6*) and are hydrolyzed by carbapenemases, which have many families, including the metallo-β-lactamases (*7*). The most recently described *bla*_NDM-1_ (New Delhi metallo-β-lactamase) is plasmid-mediated metallo-β-lactamase and has been isolated from the environment and from hospitals (*14*).

## Conclusions

We isolated a clinical strain of *V. cholerae* producing an AmpC β-lactamase and a carbapenemase. Our findings, although perhaps not an issue for treatment of cholera, have other implications. The critical role of all bacteriology laboratories needs to be emphasized for determining not only resistance patterns but also the mechanisms of resistance. Health care–associated networks need to be strengthened to ensure justified and appropriate use of antimicrobial agents that will result in safe drinking water and improved sanitation; these can have remarkable effect in reducing the spread of many communicable diseases, such as cholera, and can go a long way in controlling the growing menace of antimicrobial drug resistance. In light of the above findings, the antimicrobial drug profile of organisms, such as *V. cholerae*, needs to be under constant surveillance in the community.
